# The bridge between cognition and behavior in acquired brain injury: A graph theoretical approach

**DOI:** 10.1002/brb3.1208

**Published:** 2019-02-06

**Authors:** Frank Jonker, Wouter Weeda, Kim Rauwerda, Erik Scherder

**Affiliations:** ^1^ Vesalius, Centre for Neuropsychiatry GGZ Altrecht Woerden The Netherlands; ^2^ Faculty of Behavioral and Movement Sciences, Section Clinical Neuropsychology VU Universiteit Amsterdam Amsterdam The Netherlands; ^3^ Department of Methodology and Statistics Leiden University Leiden The Netherlands

**Keywords:** behavior, brain Injury, executive functions, frontal lobe, graph theory

## Abstract

**Background:**

The assumption is that executive dysfunctions (EF), associated with frontal lobe injury, are responsible for behavioral disturbances. Some studies do not find a relationship between EF and behavior following frontal lobe lesions. Our main goal of this study was to use a novel statistical method, graph theory, to analyze this relationship in different brain injury groups; frontal lobe damage, non‐frontal lobe damage, and controls. Within the frontal group, we expect to find a pattern of executive nodes that are highly interconnected.

**Methods:**

For each group, we modeled the relationship between executive functions and behavior as a network of interdependent variables. The cognitive tests and the behavioral questionnaire are the “nodes” in the network, while the relationships between the nodes were modeled as the correlations between two nodes corrected for the correlation with all other nodes in the network. Sparse networks were estimated within each group using graphical LASSO. We analyzed the relative importance of the nodes within a network (centrality) and the clustering (modularity) of the different nodes.

**Results:**

Network analysis showed distinct patterns of relationships between EF and behavior in the three subgroups. The performance on the verbal learning test is the most central node in all the networks. In the frontal group, verbal memory forms a community with working memory and fluency. The behavioral nodes do not differentiate between groups or form clusters with cognitive nodes. No other communities were found for cognitive and behavioral nodes.

**Conclusion:**

The cognitive phenotype of the frontal lobe damaged group, with its stability and proportion, might be theoretically interpreted as a potential “buffer” for possible cognitive executive deficits. This might explain some of the ambiguity found in the literature. This alternative approach on cognitive test scores provides a different and possibly complimentary perspective of the neuropsychology of brain‐injured patients.

## 1 INTRODUCTION

Acquired brain injury (ABI) is a broad term to indicate brain injury after birth with different etiology, not including degenerative disorders such as Alzheimer's, Parkinson's, or Huntington's disease. The general view is that ABI is strongly related to cognitive and behavioral dysfunction (Hanna‐Pladdy, 2007). More specifically, the relationship between cognitive dysfunctions and changes in behavior is most evident following injury in the frontal lobe (Wallesch, Curio, Galazky, Jost, & Synowitz, 2001). The assumption is that in particular executive dysfunctions (EF), known to be associated with frontal lobe injury, are responsible for behavioral disturbances (Alvarez & Emory, 2006; Barkley, 2001; Reid‐Arndt, Nehl, & Hinkebein, 2007). These cognitive and behavioral changes after frontal lobe damage are also paraphrased as a “dysexecutive syndrome” (Chan, 2001). Despite the strong evidence between structure and functions, there are also studies that find no relationship between executive functions and behavior following frontal lobe lesions (Blair & Cipolotti, 2000; Fujii et al., 2005; Namiki et al., 2008). The question arises why some behavioral problems after acquired brain injury of the frontal cortex do not correlate with executive dysfunction. One explanation might be that many executive functions tests lack real‐life or ecological validity, as during the traditional administration of neuropsychological tests the examiner provides structure, organization, guidance, planning, and monitoring necessary for optimal performance (Gioia & Isquith, 2004). This fails to induce executive behavioral deficits in daily live.

The effect of focal brain lesions can alter cognitive dysfunctions in multiple domains and may therefore be best understood in the context of functional networks (Gratton, Nomura, Pérez, & D'Esposito, 2012; Woolgar, Bor, & Duncan, 2013). For example, performance on the Wisconsin Card Sorting Test (WCST), one of the most distinctive prefrontal lobe tests for over the last decades, has not only been associated with frontal lobe damage (Goldstein, Obrzut, John, Ledakis, & Armstrong, 2004) but also with damage to the temporal cortex, more specific the hippocampus (Giovagnoli, 2001; Rzezak et al., 2009) and non‐frontal regions (Leskelä et al., 1999) such as subcortical and cerebellar regions (Mukhopadhyay et al., 2008). There is a general consensus that cognition is highly distributed and depends on the interaction between many brain regions (Gläscher et al., 2012; de Haan et al., 2009; Mukhopadhyay et al., 2008).

The standard approach to analyze differences in executive functioning between ABI patients and controls is to compare differences in *mean* performance on EF tests across different injury and control groups. Differences in performance between groups are then interpreted as certain cognitive systems being affected by the brain injury. This approach does not take into account the correlations between the different EF tests, and whether this pattern of correlations is different across groups. Information on the correlations between EF tests might give more insight in, for example, compensatory mechanisms, where nonlesion brain regions take over or compensate for lesion regions. In this case, a lesion in one brain area might not trigger a difference in mean EF performance, but might change the interrelations between EF tests. Focusing on the correlations between EF tests might thus give researchers additional information on EF in relation with brain injury over just mean performance. The fact that some studies do not find mean differences in frontal brain injury groups on EF tests does not mean that there are no correlation differences these groups do not differ in the pattern of interrelations between these variables. The use of the standard statistical approach might therefore be, to some extent, limited in elucidating the relation between frontal lobe damage, executive dysfunctions, and behavior, since it neglects the intercorrelations between other cognitive functions conjointly.

The current view is that localized brain regions support cognition and behavior has gradually given way to the realization that it is about connectivity (Bullmore & Sporns, 2009; Park & Friston, 2013). In recent years, functional connectivity changes associated with brain injury have been studied using the mathematical concepts of graph theory (Caeyenberghs, Verhelst, Clemente, & Wilson, 2017). Graph theory provides a method to study the relation between network structure and function. It provides a method to evaluate functional connectivity patterns, using fMRI, between brain areas (Betzel & Bassett, 2017; Bullmore & Sporns, 2009; Goñi et al., 2014). There are only a few studies that used graph theory to assess functional connectivity after traumatic brain injury (TBI) (Caeyenberghs et al., 2017). Some do report a correlation between specific networks and behavioral and clinical measure in TBI and conclude that the disturbed network can explain cognitive dysfunctions (Caeyenberghs et al., 2012; Fagerholm, Hellyer, Scott, Leech, & Sharp, 2015; Nomura et al., 2010).

An eloquent approach is applying concepts from graph theory to cognitive functions in different location of lesions following ABI. In this integrative approach, tests are explicitly modeled as a network of interrelated variables and the properties of this network are interpreted instead of analyzing tests separately (Borsboom & Cramer, 2013; Cramer, Waldorp, van der Maas, & Borsboom, 2010; Schmittmann et al., 2013) and have proven a very effective and informative way to explore brain networks and human behavior (Bassett et al., 2009; Opsahl, Agneessens, & Skvoretz, 2010).

Network analyses are different from, for example, regression approaches where there is a clear distinction between outcome and predictor variables. The network approach contains only predictors and looks for a sparse set of predictors that explain a reasonable amount of variance, that is, an optimal set of predictors that still describes the important relationships between all predictors. In that sense, it is also different from factor analytic approaches as these approaches aim for a different criterium: maximum amount of variance explained. Network approaches search for a trade‐off between explained variance and number of predictors.

Our main goal of this study was to use this alternative approach in different ABI patients groups (frontal lobe damage, non‐frontal lobe damage en controls) and provide a different and arguably complimentary perspective on the relationship between cognitive dysfunctions and changes in behavior following injury in the frontal lobe. First of all, the examination of cognitive network topology using graph theory will yield visual insights into the nature of the (dis)‐organization of cognitive structure in the different groups. Within the frontal group, we expect to find a pattern of relations between the EF tests, that is, clusters of cognitive nodes are highly interconnected with each other but not with other clusters. This cognitive phenotype of the frontal lobe group might give insight, for example, if there is indication for a possible compensatory mechanism.

Results of this explorative study may give insight into the interrelations between executive functions and behavior in relation to the different locations of lesions in a group of ABI patients. To our knowledge, this is the first study that uses a graph network theoretical approach to compare different outpatients groups with ABI on standard neuropsychological and a behavioral by using a networks approach within three groups.

## 2 MATERIALS AND METHOD

### 2.1 Participants

A total of 127 patients and 67 controls were recruited from the mental health institute for Neuropsychiatry, Vesalius, a sub‐department of mental health institute Altrecht, the Netherlands. All patients were outpatients that were referred to this institute because of neuropsychiatric, social, and/or neuropsychological difficulties, which are expected to be a consequence of acquired brain injury. The control group are patients with cognitive complaints and were randomly selected based on no structural damage and a complete neuropsychological battery. None of the controls’ subjects met the criteria of mild traumatic brain Injury (mTBI), there was no posttraumatic amnesia (PTA) or loss of consciousness (LOC). None of the controls showed structural abnormalities on the MRI. All participants were grouped into three groups; frontal lobe damage (*N* = 61), patients with non‐frontal lesions (*N* = 66), and controls with no lesions (*N* = 67). Level of education was determined by a 7‐point scale: (a) uncompleted elementary school; (b) 6 grades of elementary school; (c) 7th and 8th grade of elementary school (d) 3 years of lower general secondary education; (e) 4 years of lower general secondary education; (f) pre‐university education and higher vocational education; (g) University and technical college (Verhage, 1964). Mean time post‐injury of the non‐frontal group is 13.9 years (*SD* = 13.6) and of the frontal group 12.4 years (*SD* = 12.3). Etiology of the lesion corresponded to stroke (32.8%), traumatic brain injury (TBI) (49.2%), removal of benign primary tumor (15.6%), other etiologies including hypoxia, cerebral encephalitis, chronic toxic encephalopathy (CTE) (13.3. %). All MRI's were made between 2008 and 2015; mean year in which the MRI is made is 2012 (*SD* ± 1.2 year). See Table 1. None of the patients reported a history of premorbid chronic psychiatric disorders, neurodegenerative pathology, or severe drug addiction. All patients had sufficient command of the Dutch language. In the non‐frontal group, 19.7% had multiple etiologies, for the frontal group this is 6.6%. Therefore, the total added number is more than 100%.

**Table 1:** Demographic characteristics, etiology and lesion location for all patients, and the frontal and non‐frontal group (M ± *SD*)

	Total sample *N* = 195	Controls *N* = 67	Non‐frontal Lesions *N* = 66	Frontal lobe Lesions *N* = 62	*p*‐value
Age *y*	43.4 ± 13.8	41.1 ± 13.1	47.2 ± 13.7	41.6 ± 13.8	0.016
Level of education	4.8 ± 1.0	4.8 ± 1.2	4.8 ± 0.8	4.9 ± 1.0	0.521
Time since injury *y *(*N* = 125)	13.2 ± 12.9	–	13.9 ± 13.6	12.4 ± 12.3	0.513
Sex (% female)	33.3	31.3	37.9	31.1	0.698
Stroke (%)	32.8	–	38.8	26.2	0.132
TBI (%)	49.2	–	35.8	63.9	0.002
Tumor (%)	15.6	–	17.9	13.1	0.457
Other etiologies (%)	13.3	–	19.7	6.6	0.033

Gender (*χ* ^2^(2) = 728, *p *= 0.698) and level of education (*χ* ^2^(12) = 11.09, *p* = 0.521) did not differ significantly between groups. No difference was found between time since injury between the frontal and non‐frontal group (*p* = 0.513). There was a significant effect for age between the three conditions (*F *[2, 192] = 4.22, *p* = 0.016). Post hoc comparisons using the Tukey HSD test indicated that the mean age of the non‐frontals was significantly different (*M* = 47.26, *SD* = 13.68, *p *= 0.025) from the controls (*M* = 41.23, *SD* = 13.16). A trend was found for age between the non‐frontal and frontal lobe group (*p* = 0.051).

### 2.2 Materials and procedure

The existing framework of the standard diagnostic procedures of Mental Health Institute Altrecht (Neuropsychiatry, Vesalius) was used. The neuropsychologist administered the neuropsychological tests and questionnaires. Demographic information and injury‐related information were collected from the electronic patient file. All tests and questionnaire have been chosen to provide an added value within the population of care. A full report was written for regular care, and a copy was provided to the patient. If a recent MRI has been made elsewhere, no additional MRI was requested. In all other cases, a new MRI was made in the University Medical Center, Utrecht (UMCU), the Netherlands on 3.0 Tesla MRI machine (Philips NT). For most patients, sagittal slices with T1‐SE sequence, transversal slices with T2‐FLAIR sequence, and coronal slices with T1‐IR and T2‐FFE sequences were acquired. A radiologist carried out the anatomic classification and etiology (where possible) of patients’ lesions. Patients were classified to the frontal lobe group if the radiologist assigned visible tissue loss, atrophy, or postoperative lesion and white matter lesions to the frontal lobe. Written informed consent was obtained from participants according to the Declaration of Helsinki, and a local ethic committee approved the study.

### 2.3 Cognitive measurements

#### 2.3.1 Working memory

Digit‐Span backwards is a subtest of the Wechsler Adult Intelligence Scale‐III (WAIS‐III‐R; (Wechsler, 1997)) and measures working memory (Baddeley, 2003), and the ability for updating and manipulate relevant information. These aspects rely mostly upon frontal lobe functioning (Gläscher et al., 2012). The task requires participants to repeat digit sequences that are presented forward and backward. We used the total number of correct items backward as a measure for working memory.

#### 2.3.2 Memory

The 15‐Word Learning Test (15‐WLT) is a measure for immediate and delayed memory and is a Dutch version of the Rey Auditory Verbal Learning Test (RAVLT) (Rey, 1964). The total correct words remembered on the immediate recall of the RAVLT (range between 0 and 75), and the number of correct words on the delayed recall of the RAVLT was used as a measures for memory.

#### 2.3.3 Executive functions

The Stroop Color‐Word Test (SCWT) measures speed of information processing and the capacity to suppress automatic response tendencies (Alvarez & Emory, 2006). An interference measure is calculated by taking the time on Stroop III divided by Stroop II (STROOP III/II), with higher ratio scores reflecting greater interference.

The Trail Making Test (TMT A,B) measures divided attention (Reitan & Herring, 1985). TMT‐A requires an individual to draw lines sequentially connecting 25 encircled numbers distributed on a sheet of paper. Part B (Trail B) is considered a measure of cognitive flexibility, alternating attention (e.g., 1,A,2,B,3,C.), and ability to inhibit a dominant but incorrect response (Kortte, Horner, & Windham, 2002). Calculating the ratio between Part B and Part A (Trails B/Trails A) is suggested for interpretation of executive deficits and eliminating the influence of visual and motor abilities on performance (Corrigan & Hinkeldey, 1987). Higher ratio scores reflect more cognitive inflexibility.

Letter fluency (DAT) is a phonemic memory task that requires patients to say as many words as possible beginning with a specific letter (the letters D, A, T are provided). This test mainly measures switching to another letter or category group and is frequently impaired after frontal lobe damage (Reverberi, Laiacona, & Capitani, 2006). Participants are instructed not to use people's names, places, and numbers or to name sequences of words with the same prefix (e.g., superman, supercars, and supermarket). Letter fluency performance is based on the number of correct items produced by the participants. Items were counted as correct if they met the constraints of the condition and were not repetitions. The total number of correct words was used in our analysis.

### 2.4 Behavioral assessment

Frontal Systems Behavioral Scale (FrSBe): The FrSBe is a 46‐item rating scale designed to measure behavioral changes after frontal lobe damage. The FrSBe includes a total score, which is a composite of three subscales: Apathy, Disinhibition, and Executive Dysfunction (Stout, Ready, Grace, Malloy, & Paulsen, 2003). In our study, patients were asked to rate each question on a five‐point Likert scale. Because continuous scores are required for carrying out network analysis, we used raw scores instead of categorical scores.

### 2.5 Data analyses

#### 2.5.1 Standard analysis

For between‐group comparisons, an ANOVA is used. For the nonnormal data, a Kruskal–Wallis test is used. Analyses were performed using IBM SPSS Statistics 22.0 (IBM Corp., Armonk, New York).

#### 2.5.2 Network analysis

In graph theory terminology, the variables are termed “nodes” and nodes are connected with each other via “edges”. Edges can be binary, that is, present or absent. They can be weighted; an edge with a higher weight is more strongly connected with a node than an edge with a lower weight. Finally, edges can be directed, that is, the edge between node A and B can be different from the edge between node B and A (Opsahl et al., 2010). In our case, the cognitive functions and behavioral measures constitute the nodes of a network with the partial correlations between the cognitive functions and behavioral measures as weighted and undirected edges.

There are several properties that can be inferred from a network, both on the network as whole, as well as on the individual nodes and edges. Widely used properties are centrality and modularity. Centrality determines how “central” a node is within a network, that is, if the node is connected to many other nodes (degree centrality) (Opsahl et al., 2010). Another way to examine a network is to look if and how different nodes cluster together into different “communities”. That is, a community can be identified as a group of nodes that are highly connected with nodes within the same community but have few connections with nodes from other communities. One effective method to examine whether there are different communities within a network is the use of “modularity”. Modularity can be defined as a measure of strength of a division of a network into “modules”. Networks with high modularity contain clusters of nodes that are highly connected with other nodes but less with nodes from other clusters (Newman, 2006a).

#### 2.5.3 Network analysis: construction of the frontal and non‐frontal network

For each group, the cognitive tests (Stroop, WM, TMT, RAVLT, DAT) and the behavioral questionnaire (FrSBe) constituted the nodes of the network. Networks were estimated within each group using the graphical LASSO (Friedman, Hastie, & Tibshirani, 2008). This procedure estimates the (inverse) partial covariance matrix of a set of variables, penalizing small covariances (i.e., setting small covariances to zero). The optimal strength of the penalty term (controlling the amount of penalty applied) was estimated using the BIC (Schwarz, 1978). The resulting partial covariance matrix thus contains only those edges that are deemed important, as all small edges are set to zero. Outcome of this analysis is thus a “sparse” matrix containing the most important partial correlations (correlations between two nodes corrected for the correlation with all other nodes in the network) between nodes.

Networks were estimated in R (R Core Team, 2016) using the *glasso* (Friedman, Hastie, & Tibshirani, 2011), *bootnet,* and *qgraph* packages (Epskamp, Cramer, Waldorp, Schmittmann, & Borsboom, 2012). We used the bootstrap version of the EBICglasso function with the tuning option set to 0, which equals model selection using the BIC (Schwarz, 1978) with 1,000 bootstraps. To visualize the network, we used a circular plot and a spring‐embedded plot. In a circular plot, the nodes are located at equal path length around a circle. Nodes that are highly connected have a high density of lines. With a spring‐embedded algorithm, nodes are located in such a way as to put those with smallest path lengths (i.e., higher correlation) to one another closest in the graph. In this procedure, randomly placed nodes are sorted into a desirable visual presentation (symmetry, nonoverlapping etc.) (Battista, Eades, Tamassia, & Tollis, 1994). To identify the between‐group differences in network measures, we investigated the nodal characteristics of the constructed networks. We calculated nodal degree centrality of each test for the three group networks. The bootstrap procedure was applied to estimate the stability of the centrality estimates. The resulting distribution of centrality values for each node was then used to calculate the 95% confidence interval (between the 2.5% and 97.5% percentile of this distribution) of the centrality values. Nonoverlap in confidence intervals between groups was taken as evidence for a difference in centrality between groups for that node. Note that bootstrap confidence intervals for centrality measures can be unreliable and must be interpreted with some caution (Epskamp, Borsboom, & Fried, 2017).

#### 2.5.4 Community analysis

The subsets of nodes that are densely connected to each other but less with other nodes are referred to as communities or clusters (Boccaletti, Latora, Moreno, Chavez, & Hwang, 2006). One effective method to examine whether there are different communities within a network is the use of “modularity”. Modularity was calculated using the Louvain algorithm for undirected and weighted graphs (Rubinov & Sporns, 2010). This method does not require the setting of a threshold of which (partial) correlations to include in the analysis. To avoid issues with local minima, we ran the algorithm 100 times and chose the solution with the highest modularity index Q (Q ranges from 0 to 1, with 1 indicating perfect separation into different modules (Newman, 2006b). To allow assessment of the stability of the detected communities, we performed the community detection algorithm to the 1,000 bootstrap samples from the network analysis. This leads to 1,000 community assignments for each node in the network. We visualized the stability of the communities by looking, for each pair of nodes, in how many of the bootstrap samples both nodes were in the same community. We visualized our data by a panel of color codes for each node corresponding to the proportion of bootstraps in which this node was in the same community as the other nodes in the network. This analysis is performed in each of the three groups separately.

## 3 RESULTS

### 3.1 ANOVA

First, we performed an ANOVA between the three groups for cognitive and behavioral measures. Analyses show no difference between groups for the cognitive measures. The assumption of homogeneity of variance for Digit‐Span backwards, RAVLT, DAT, Stroop is found tenable using Levene's test (all *p*'s > 0.05). No significant difference is found for Digit‐Span backwards; *F*(2,192) = 1.113, *p* = 0.331; RAVLT ‐ total, *F*(2, 191) = 2.162, *p* = 0.118; RAVLT‐recall, *F*(2, 191) = 2.832, *p* = 0.061; DAT, *F*(2,188) = 0.166, *p *= 0.847; and Stroop *F*(2,185) = 0.173, *p *= 0.841). For the TMT, the assumption of homogeneity of variance was tested using a nonparametric Levene's test and found tenable (*p* > 0.05). A Kruskal–Wallis test shows that there is no statistically significant difference between groups for TMT *χ* ^2^ (2, *N* = 195) = 2.52, *p* = 0.283. The assumption of homogeneity of variance for FrSBe is found tenable for all three subscales using Levene's test (all *p*'s > 0.05). A significant difference is found for apathy *F*(2,192) = 5.308, *p* = 0.006 and for Executive Dysfunction *F*(2, 192) = 5.106, *p* = 0.007. Post hoc analysis using Turkey HSD indicates that the mean score on the Apathy subscale of the control group is significantly higher (40.87) compared to the non‐frontal group (33.11). Post hoc analyses for Executive Dysfunction using Turkey HSD show a significantly higher score for the control group (48.25) compared to the non‐frontal group (42.06).

### 3.2 Visualization of the networks

**Figure 1:**
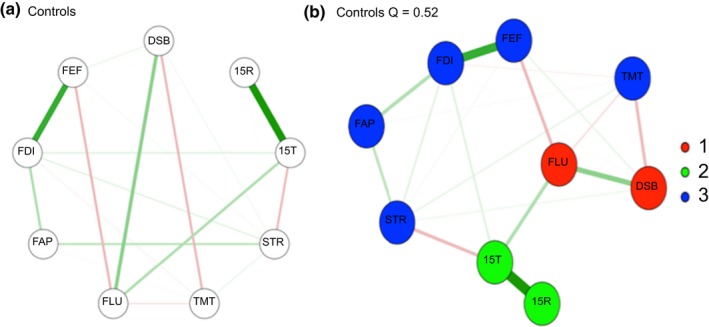
(a) Circular plots Controls. (b) spring plots controls. 15R: Word Learning Test Recall score; 15T: 15‐Word Learning Test Total score; DSB: Digit‐Span backwards; FAP: FrSBe‐Apathy scale; FEF: FrSBe‐Executive Dysfunction scale; FID: FrSBe‐Disinhibition scale; FLU: letter fluency; STR: Stroop Word‐Color test; TMT: Trail Making Test. Cluster 1‐green) memory (15T‐15R); Cluster 2‐red) Letter fluency and working memory; Cluster 3‐blue) Stroop, divided attention all FrSBe subscales

**Figure 2:**
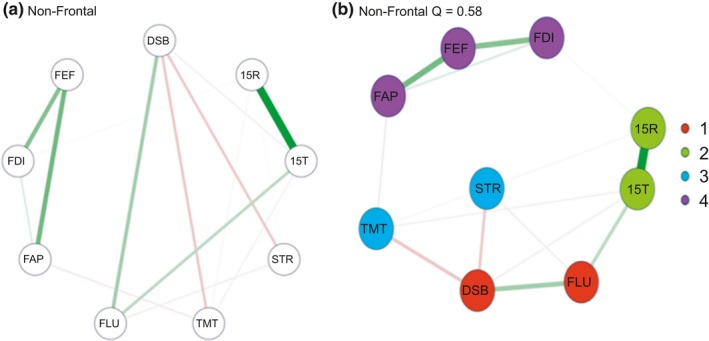
(a) Circular plots Non‐Frontal. (b) spring plots Non‐Frontal. 15R: Word Learning Test Recall score; 15T: 15‐Word Learning Test Total score; DSB: Digit‐Span backwards; FAP: FrSBe‐Apathy scale; FEF: FrSBe‐Executive Dysfunction scale; FID: FrSBe‐Disinhibition scale; FLU: letter fluency; STR: Stroop Word‐Color test; TMT: Trail Making Test. Cluster 1‐green) memory (15T‐15R); Cluster 2‐red) Letter fluency, Working Memory; Cluster 3‐purple) all FrSBe subscale; Cluster 4‐blue) stroop and divided attention

**Figure 3:**
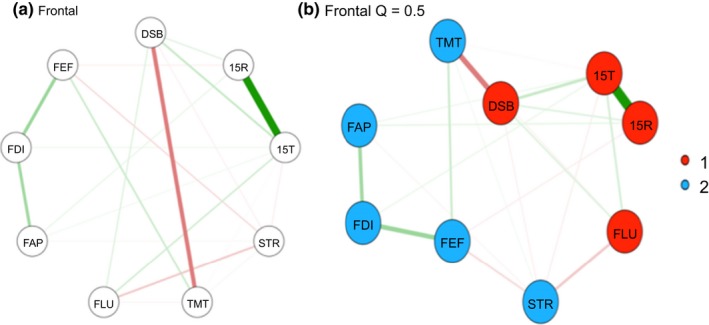
(a) Circular plots Frontal. (b) spring plots Frontal. 15R: Word Learning Test Recall score; 15T: 15‐Word Learning Test Total score; DSB: Digit‐Span backwards; FAP: FrSBe‐Apathy scale; FEF: FrSBe‐Executive Dysfunction scale; FID: FrSBe‐Disinhibition scale; FLU: letter fluency; STR: Stroop Word‐Color test; TMT: Trail Making Test. Cluster 1‐red) memory (15T‐15R), letter fluency and working memory; Cluster 2‐blue) Stroop, divided attention and all FrSBe subscales

All networks are represented in Figures 1a,b, 2a,b, 3a,b (a; circular; b; spring plots). The nodes (circles) represent the different neuropsychological tests and behavioral questionnaires. The lines between nodes represent the interrelations between those measures (partial correlations). The width of the lines indicates the strength of the correlation; the red and green lines represent a negative (red) or positive (green) partial correlations. Different colors indicate to which community the nodes belong (see next section). In all three different spring plots, a different cognitive measure seems to be the most central node; for controls letter fluency (FLU); for non‐frontal the Stoop (STR); and for the frontal group working memory (DSB) and executive behavior (FEF). Of interest is that the spring plots of the non‐frontal group have fewer interconnections and therefore seem to be the least complex network compared to the non‐frontal and frontal group. In all networks (spring and circular), the nodes 15T and 15R have a strong positive connection.

### 3.3 Modularity estimation

In the control group (Figure 1b), there are three clusters. Cluster 1‐green) memory (15T‐15R); Cluster 2‐red) Letter fluency and working memory; Cluster 3‐blue) Stroop, divided attention and all FrSBe subscales. In the non‐frontal (Figure 2b) group, there were four clusters. Cluster 1‐green) memory (15T‐15R); Cluster 2‐red) Letter fluency, Working Memory; Cluster 3‐purple) all FrSBe subscale; Cluster 4‐blue) Stroop and divided attention. In the frontal group (Figure 3b), there are only two clusters. Cluster 1‐red) memory (15T‐15R), letter fluency and working memory; Cluster 2‐blue) Stroop, divided attention and all FrsBe subscales. In all the networks, working memory and letter fluency form a cluster, in the frontal group is it forms a cluster with all the subscales of the FrSBe.

### 3.4 Degree centrality

**Figure 4:**
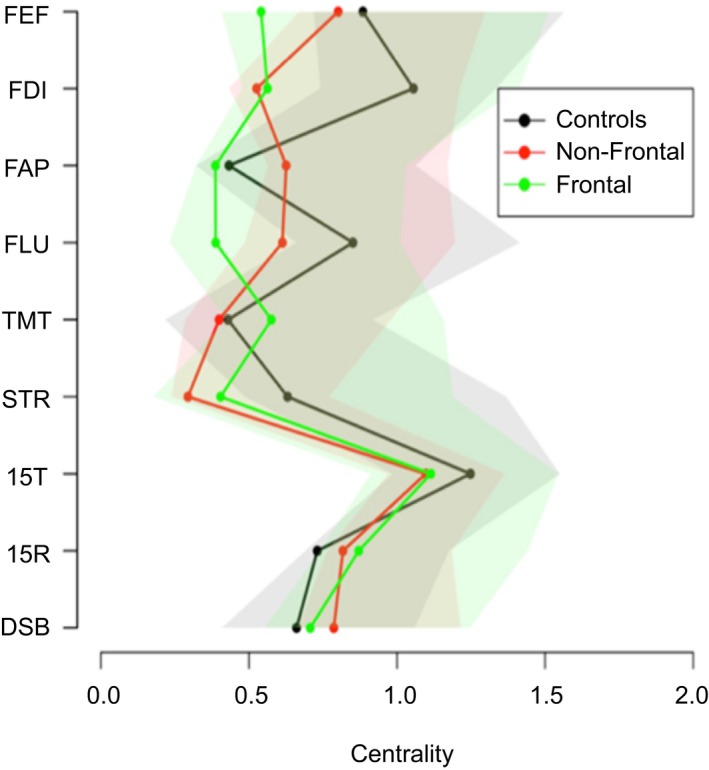
Degree centrality between groups. Colored areas around the lines represent 95% confidence interval the boostrap distribution. 15R: Word Learning Test Recall score; 15T: 15‐Word Learning Test Total score; DSB: Digit‐Span backwards; FAP: FrSBe‐Apathy scale; FEF: FrSBe‐Executive Dysfunction scale; FID: FrSBe‐Disinhibition scale; FLU: letter fluency; STR: Stroop Word‐Color test; TMT: Trail Making Test

In order to investigate the importance of the cognitive tests and the behavioral questionnaires within each group's network, we calculated the degree centrality between groups. The degree centrality of a node reflects how strong this node is connected to the rest of the network. Degree centrality between groups is illustrated in Figure 4. No difference in centrality was found between groups for the cognitive and behavioral nodes.

**Figure 5:**
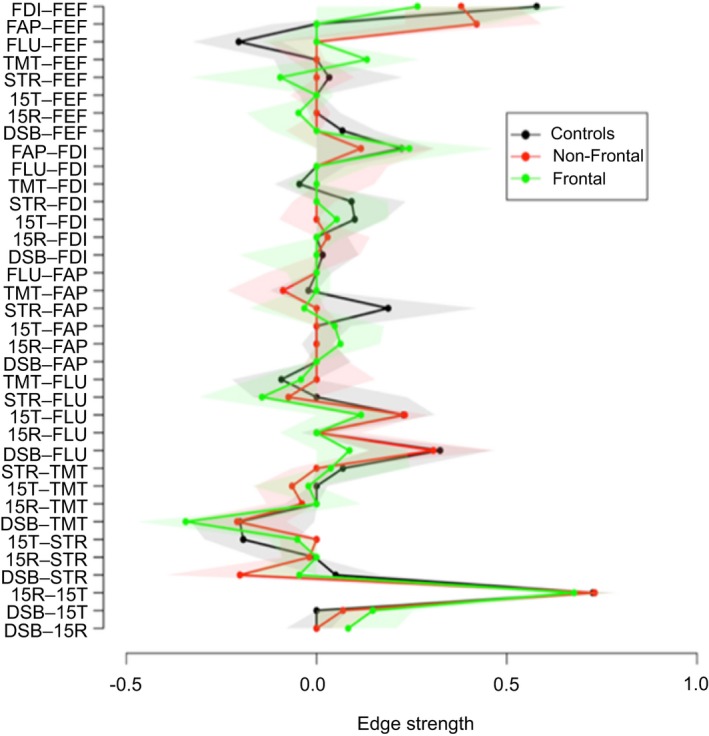
Edge weights between tests. Colored areas around the lines represent 95% confidence interval the bootstrap distribution. 15R: Word Learning Test Recall score; 15T: 15‐Word Learning Test Total score; DSB: Digit‐Span backwards; FAP: FrSBe‐Apathy scale; FEF: FrSBe‐Executive Dysfunction scale; FID: FrSBe‐Disinhibition scale; FLU: letter fluency; STR: Stroop Word‐Color test; TMT: Trail Making Test

Figure 5 shows the weight of all pairs of non‐zero edges in the networks of all groups. Of interest is that the DSB and TMT seem to have less weight in the frontal group. FDI–FEF and STR–FAP seem to have a strong weight within the control group. FLU–FEF has a low weight in the control group. For the non‐frontal group, a large weight is found between FAP‐FEF and 15T‐FLU, a low weight for DSB‐STR. For all groups, there is a high weight between 15T‐15R and DSB‐FLU.

### 3.5 Community analysis

**Figure 6:**
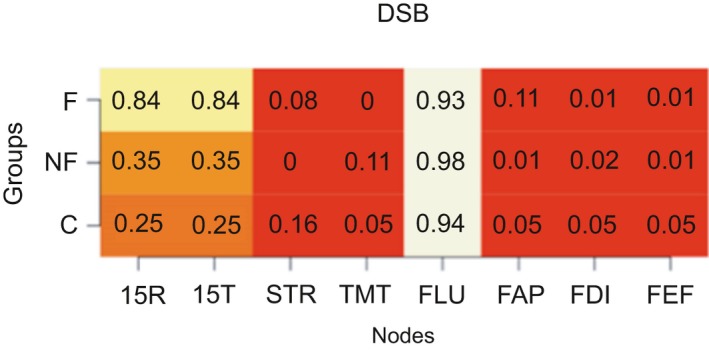
Digit symbol backwards

**Figure 7:**
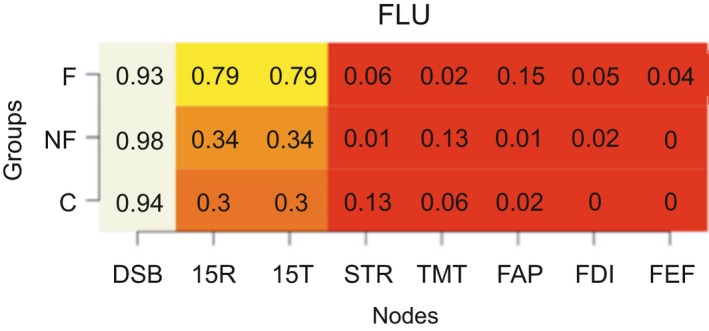
Letter fluency

**Figure 8:**
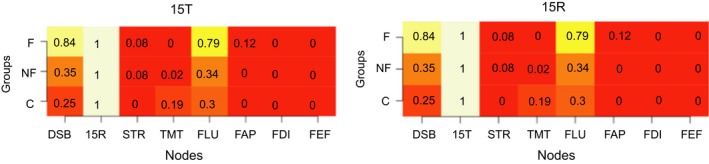
15WT‐Total & 15WT‐Recall

**Figure 9:**
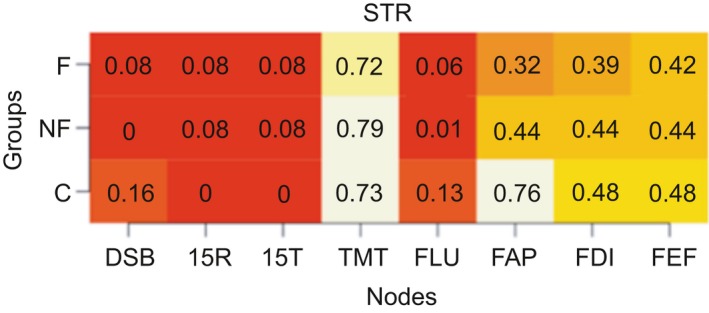
Stroop

**Figure 10:**
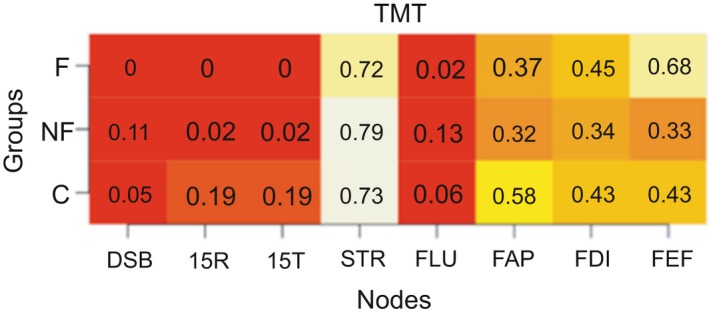
Trail Making Test

**Figure 11:**
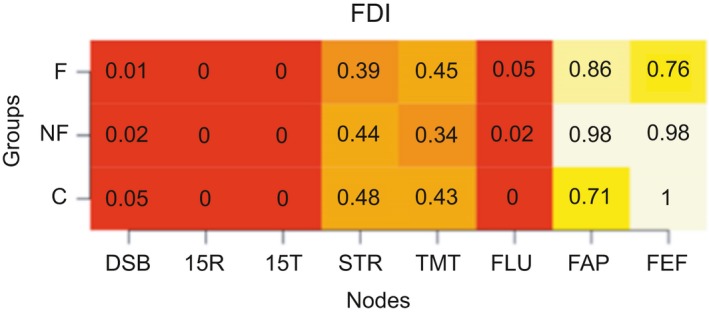
FrSBe disinhibition

**Figure 12:**
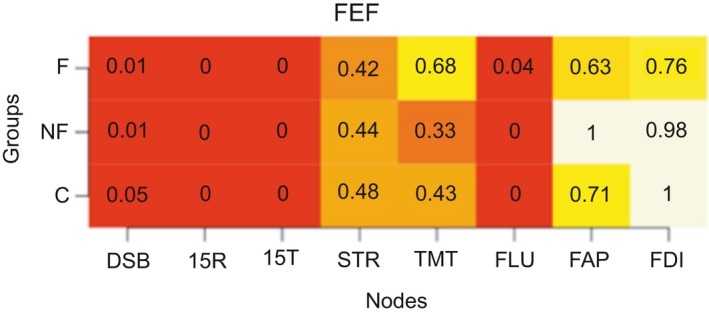
FrSBe executive functions

**Figure 13:**
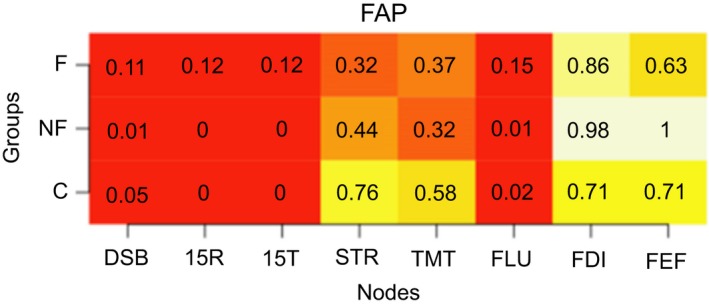
FrSBe apathy. 15R: Word Learning Test Recall score; 15T: 15 Word Learning Test Total score; DSB: Digit‐Span backwards; FAP: FrSBe‐Apathy scale; FEF: FrSBe‐Executive Dysfunction scale; FID: FrSBe‐Disinhibition scale; FLU: letter fluency; STR: Stroop Word‐Color test; TMT: Trail Making Test

We calculated the modularity index Q, which ranges from 0 to 1, with 1 indicating perfect separation into different modules and 0 indicating community assignment is on chance level. The absolute stability of the detected communities is reflected in a high proportion, indicating in what proportion of the bootstrap samples the node of interest is in the same community as another node (separate columns). The differentiation between communities is reflected in the differences between the proportions in the rows (i.e., the three groups) for each column. Proportions of “same‐community‐assignment” for each node are in Figures 6, 7, 8, 9, 10, 11, 12, 13, one figure for each node of interest. The rows indicate the three groups, while the columns indicate all the nodes besides the node of interest. Proportions are given as numbers in each cell. All cells are also color‐coded to reflect the proportion (white = high proportion, red = low proportion). The difference between “same‐community‐assignment” proportions *across* groups thus indicates that the node of interest “clusters” with different EF tasks across groups, an indication that the pattern of interrelations is therefore different across the groups. Vice‐versa, a similar pattern of “same‐community‐assignment” proportions across groups indicates that the node of interest forms a community with the other nodes in a similar pattern. We did find different proportions in the community assignments of nodes between groups. Figures 6, 7 show that DSB (84%) and FLU (79%) form a community with 15T and 15R in the frontal group but not in the control (25%) and non‐frontal (35%) groups. DSB and FLU form a community over all groups and do not differentiate between groups (frontal = 93%, non‐frontal = 98%, control = 94%). A large proportion is found between STR and TMT varying from 73% to 79% in all groups. A proportion of 76% is found for FAP and STR in the control group, whereas in the non‐frontal and frontal groups, these proportions were 44% and 32% (Figure 9). A proportion of 68% was found for FEF and the TMT (Figure 12) in the frontal group with lower proportions for the control (43%) and non‐frontal (33%) groups.

## 4 DISCUSSION

In this explorative study, we applied graph analysis (i.e., networks) on different tests of executive functioning and behavioral measures within three groups (a) frontal lesions, (b) nonfrontal brain damage, and (c) controls. We found that the three groups have different cognitive phenotypes and that they are marginally related to behavioral measures. We did not find any mean differences (ANOVA) between the groups for cognitive measures. Conform literature, despite the fact that the frontal lobe group had more structural damage (tissue loss, atrophy, postoperative lesion, white matter lesions), we did not find more executive dysfunctions (Fujii et al., 2005; Namiki et al., 2008). The frontal group did not report significantly more behavioral problems. For the FrSBe, we did find a significant difference (ANOVA) for the controls for Apathy and Executive Dysfunction compared to the non‐frontal group. Besides, it has been shown that self‐reported FrSBe scores in a healthy control group (*n* = 127) showed significant differences in executive dysfunctions and was a predictor of behavioral problems (e.g., credit card debts) (Yang, Spinella, & Lester, 2004). Other studies also show elevated score on the FrSBe in healthy controls (Pluck et al., 2012).

A less expected finding was that we did not find any differences in centrality between groups for the cognitive and behavioral nodes in de frontal lobe group. Based on our analysis, we found that the total performance of the immediate free recall (15T) has the most centrality among all tests in all the groups, that is, that other cognitive functions strongly associated with immediate verbal memory. Its connection with 15R is strong in all groups. Both 15R and 15T form a community in 100% (Figure 8) of the bootstrap samples for all groups, meaning that 15T and 15R do not differentiate between groups. It forms a community with working memory (DSB) and letter fluency (FLU) in the frontal lobe group, but not in other groups. Verbal memory (15T, 15R) does not form a community with the behavioral subscales, suggesting that location of lesions does not differentiate between groups for the score on verbal memory in relation to behavior. It is striking that this frontal cognitive phenotype involves merely mental processes (working memory, letter fluency, verbal memory) and does not involve tasks involving visual‐motor tasks (Trail making, Stroop). One could conclude that the different executive components, strategic retrieval processes (FLU) and monitoring (DSB), are devoted for encoding and retrieval of information. In addition, working memory (DSB) and fluency (FLU) do not differentiate between groups. In the frontal lobe group, these executive components might theoretically serve as a “compensatory mechanism” or “supporting functions” for verbal memory deficits.

The TMT and Stroop do not form communities with other cognitive variables but do seem to have a strong interrelationship due to a common processing speed element (Demakis, 2004). One of the main findings of our study is that the network approach is more able to uncover, and visualize, the interrelationships between tests scores in the frontal group in the absence of *mean* difference between groups. In line with our clinical findings, a recent fMRI study showed that focal frontal lesions showed an increased fronto‐partietal activity (e.g., parietal and ventral stream activity) compared to controls with non‐frontal lesion on different cognitive tasks. The authors suggest that the effects of focal lesions may be best understood in the context of adaptive brain‐wide cognitive network (Woolgar et al., 2013).

Recent reports have suggested frontal lobe specificity for the TMT, especially the dorsolateral prefrontal cortex (DLPFC) (Davidson, Gao, Mason, Winocur, & Anderson, 2008; Demakis, 2004). Despite its low centrality (and therefore low weights between other nodes), a stable community (e.g., high proportion) was found between the STR and TMT in all groups, suggesting a great interdependence between these functions with no differentiation between groups. This is in line with the assumption that the total score and ratio score (B/A) of the TMT are not specific or sensitive to frontal lobe damage (Goldman‐Rakic, 1987; Stuss et al., 2001). Some larger proportion in the frontal group (FEF, 68%) and control group (FAP, 58%) suggests a relationship with cognitive flexibility. TMT forms no community with working memory, letter fluency, and verbal memory in all the groups. A lack of correlation between TMT ratio and working memory is consistent with literature (Gläscher et al., 2012).

With regard to the Stroop ratio score, it is notable that it forms no communities with the FrSBe subscales in all the networks. The Stroop does also not form a community with working memory, letter fluency, and verbal memory. Despite the difference in several studies, specific frontal lobe areas are related to the performance of the Stroop, namely DLPFC and the anterior cingulate cortex (ACC), but fails to discriminate between frontal and non‐frontal lesions (Alvarez & Emory, 2006; Botvinick, Cohen, & Carter, 2004). Of interest is the fairly high differentiation of the Stroop (76%) for apathy in the control group.

We have made no distinction in this study between the various locations of lesions in the frontal lobe. This is possibly reflected in the observation that, in all networks, in each group, the subscales of the FrSBe do not differentiate between groups (i.e., community stability was equal across all groups). In all the groups, a high range of proportions is found for all subscales (63%–100%) with no clear differentiation. One could carefully conclude that frontally mediated behaviors are partially independent of cognitive performance (Stout et al., 2003).

Results of our network analysis emphasize that the relationship between executive function and behavior in a group of ABI patients, with different locations of lesions, is not straightforward. Since this is a noninferential and more hypothesis generating approach, our results could possibly be the start of the development of new brain‐cognition‐behavior theories and should lead to viable avenue of new research targeting the adaptive brain‐wide network on cognition in relation to behavior in a clinical setting.

### 4.1 Limitations

In this explorative study using a graph analyses approach with cognitive and behavioral nodes in patients with frontal/non‐frontal ABI, we agree that the number of subject within each group was relatively low to perform the graph analysis. The reasons for using a graph theoretical or network approach were mainly to give insight into the relationships between often‐used neuropsychological tests in groups with brain injury. The network approach provides a different view on these relations and can lead to hypothesis about the underlying brain mechanisms (i.e., brain regions compensating for the failure of other regions). Since the groups in our study are relatively small for a network analyses, the results still show interesting avenues for new research. For example, by performing a community structure analysis, we showed that different tests tend to cluster together across different ABI groups. The measures used in our study (i.e., centrality) and the algorithms used for community analysis are not the only ones available. Different measures might highlight different aspects, and different algorithms might give different results. We feel that it is beyond the scope of this paper to address all different methods, as our main goal is to highlight the viability of the network approach in a clinical sample of ABI patients and to generate possible new and innovative hypotheses about the underlying mechanisms of ABI.

Although we grouped our patients in non‐frontal and frontal, additional/overlapping lesions may have influenced the effect on cognitive outcomes. This is particularly true for the frontal group, which has significantly more TBI. TBI is more likely to cause not only one distinct anatomical injury but also more diffuse white matter jury, which theoretically may cause more disruption between regions of the brain. Given this is a clinical population we did not have the opportunity to make an exact distinction of the (overlapping) lesions.

Subsequently, the heterogeneity of the nature of acquired brain injury in our patient sample is high (Stuss & Levine, 2002). The cross‐sectional design of the study might have narrowed the range of possible outcomes and decreased variability among patients and controls. It did not allow us to examine the change in cognition and behavior. Another limitation is high variability in time since injury, as recovery stage might influence the test results. One can expect that patients with a more extended period of time since injury might have had more opportunities to enroll in interventions to enhance cognitive functioning or have learned to compensate for their deficits. Regarding demographic variables, the non‐frontal group is significantly older. A final limitation is the use of ratio scores; the reason for the absence of centrality between tests might be that ratio scores between two tests often have a low reliability.

### 4.2 Future work

We are aware of the explorative nature of this study and the relatively small sample size. We are therefore cautious about our statements regarding the reliability of the networks. The current study was not based on specific hypothesis but does provide insight into the underlying coherence of different modalities of cognitive functioning. This approach represents a change of perspective from the traditional analysis of mean differences toward an approach based on interrelatedness of different modalities. Despite our cautiousness, we would like to make some suggestions about the possible usability of this approach and pose some possibilities for future research. In clinical practice, the concept of executive functions is based on abnormal scores of tests, and statements are made about executive functions even if (some of) these tests are not normal. We showed that the mutual relationship between the different cognitive tests can be informative as well. The relationships between the different modalities provided insight in to which roles modalities play with different types of lesions. Based on our study, we believe that a next step in this research field could be the development of prototype networks (e.g., frontal, non‐frontal) to which an individual networks can be compared. This gives the clinician insight into the relative strengths and weaknesses of the mutual relationships of the individual test scores and functions. Based on this outcome, one might theoretically make statements about whether or not therapeutic interventions are successful. In addition, an interesting line of research would be to link these prototypical networks to clinical symptoms (e.g., irritability, reduction of initiative) instead of behavioral clusters. Last but not least, it would be highly informative to, for example, use the strength of functional brain connectivity measures in these prototypes of networks.  With this approach, you could theoretically create risk profiles for patients. The strength of the connection between brain networks and prototypical networks might put the patient at greater risk for developing psychiatric‐behavioral symptoms.

## 5 CONCLUSION

Results from this study illustrate a different perspective on the interrelations between executive functions and behavior and might contribute to the question why some behavioral problems after acquired brain injury of the frontal cortex do not co‐occur with executive dysfunction. The different cognitive and behavioral clusters, each with a different stability and proportion, might be theoretically interpreted as a potential “buffer” for possible cognitive deficits (Satz, Cole, Hardy, & Rassovsky, 2011). Future research, with well‐defined locations of lesions in different ABI groups, could identify important network characteristics, which could be a target for treatment. Our results could possibly be the start of the development of new treatment protocols that strongly rely on these different characteristics. Furthermore, this means that the standard neuropsychological protocol should be extended with multiple tests per cognitive domain (e.g., memory, attention, executive functions) in order to “capture” the effects of plasticity in an ABI outpatients group.

## CONFLICT OF INTEREST

The authors declare that they have no competing interests.

## AUTHORS' CONTRIBUTIONS

Contributions to the manuscript were the following: FAJ acquired the data and wrote the manuscript. Dr. WDW is coauthor and performed the statistical analyses. KR acquired the data and helped‐out with the manuscript. Prof. Dr. ES supervised the manuscript and gave advice.

## ETHICS APPROVAL AND CONSENT TO PARTICIPATE

Written informed consent was obtained from participants according to the Declaration of Helsinki, and a local ethic committee approved the study.

## AVAILABILITY OF DATA MATERIALS

The datasets generated and/or analyzed during the current study are not publicly available due to the fact that the data were obtained in the course of mental health care but are available from the corresponding author on reasonable request.
